# A Ticking Time Bomb: A Case of Floating Distal Aortic Arch Intraluminal Thrombus

**DOI:** 10.7759/cureus.32212

**Published:** 2022-12-05

**Authors:** Mohamed Ghoweba, Jason Gnasigamany, Madhu Chiluveri, John McClish

**Affiliations:** 1 Internal Medicine, Texas A&M College of Medicine/CHRISTUS Good Shepherd Medical Center, Longview, USA; 2 Cardiology, Texas A&M College of Medicine/CHRISTUS Good Shepherd Medical Center, Longview, USA

**Keywords:** arterial thrombosis, mural arch thrombus, mural thrombus, intraluminal thrombus, aortic arch thrombus

## Abstract

Aortic arch thrombus is a rare entity that can result in catastrophic sequelae. This is a case report of a 65-year-old female patient who presented with chest pain that started one day prior to arrival at the emergency department. Acute coronary syndrome (ACS) and pulmonary embolism (PE) were ruled out. A filling defect at the distal aortic arch evident on chest computed tomography angiography (CTA) was confirmed to be a floating distal aortic arch thrombus on transesophageal echocardiogram (TEE). There was no evidence of an underlying aneurysm, dissection, or significant atherosclerosis. The patient was considered to be at high risk for surgical intervention, hence, a decision was made to start the patient on chronic anticoagulation with direct oral anticoagulants (DOACs). A follow-up CTA three months later showed total resolution of the thrombus. The report highlights this treacherous pathology and provides an overview of the predisposing factors, radiologic findings, as well as management strategies for floating aortic arch thrombi.

## Introduction

Aortic arch thrombus without an aneurysm or dissection is an extremely rare finding with possible dire consequences. Incidence is reported to be about 0.45% [1]. The most common location was reported to be the descending aorta followed by the aortic arch, while the ascending aorta was the least reported [2]. Although the exact pathophysiological origin remains ambiguous, multiple predisposing factors have been postulated. Diagnosis is often coincidental using chest imaging modalities, however, patients can present with signs and symptoms of arterial embolization. Management is predominantly based on clinical experience with no consensus in the medical literature. Surgical intervention is often necessary, while anticoagulants have been widely successful in treating the condition.

## Case presentation

A 65-year-old Caucasian woman with a history of hypertension, coronary artery disease (CAD) status post coronary artery bypass graft (CABG) nine months prior, chronic obstructive pulmonary disease (COPD), diabetes mellitus type II not on chronic use of insulin, acquired hypothyroidism, and tobacco use disorder of half a pack a day (24 pack-years), who presented to the emergency department complaining of chest pain and shortness of breath that started one day prior to presentation.

The patient’s pain was described as retrosternal, sudden in onset, radiating to her left arm, sharp in character, 10/10 in severity, aggravated with exertion, mildly relieved at rest, and associated with shortness of breath, nausea, lightheadedness, and palpitations. The patient also complained of worsening cough and wheezing over the preceding few days. She had been compliant with her cardiac medications including oral aspirin 81 mg daily, atorvastatin 40 mg daily, amlodipine 10 mg daily, losartan 50 mg daily, metoprolol 25 mg twice daily, and levothyroxine 50 mcg daily.

**Figure 1:**
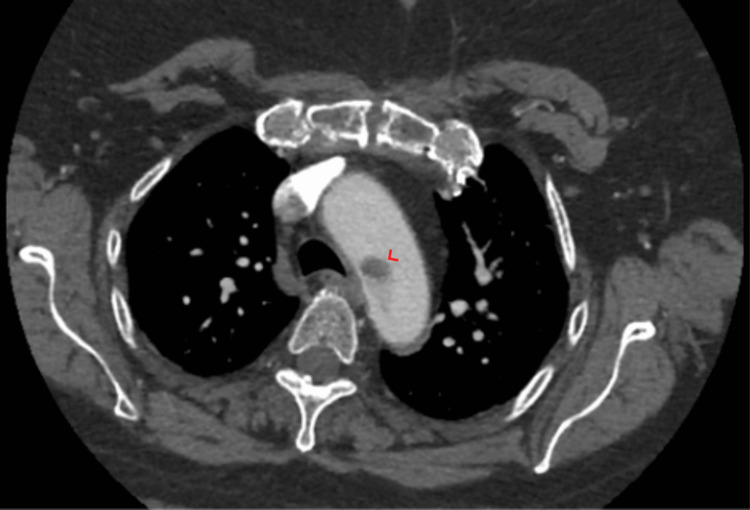
CTA/Aortogram showing a non-occlusive distal aortic arch filling defect (arrowhead). CTA: computed tomography angiogram

Vitals on arrival were unremarkable except for elevated blood pressure at 170/70 mmHg. A physical exam was evident for diffuse bilateral chest wheezes and moderate diffuse chest wall tenderness. Lab values including complete blood count (CBC) and basic metabolic panel (BMP) were unremarkable except for an elevated blood glucose level of 320 mg/dL. Troponin I level remained within normal levels at <0.03 ng/mL on three readings. Chest X-ray showed moderate pulmonary congestion. Electrocardiogram was unremarkable. D-dimer was elevated at 1255 ng/mL. Chest computed tomography (CT) following pulmonary embolism (PE) protocol showed a low-density filling defect within the aortic arch denoting a possible intraluminal thrombus. CT angiography (CTA) with and without contrast showed a non-occlusive aortic arch thrombus (Figure 1). A 2D transesophageal echocardiogram (TEE) with color flow Doppler showed a highly mobile 2 cm x 1.4 cm mass in the distal aortic arch suggestive of a thrombus (Video 1).

**Video 1:** 2-dimensional transesophageal echocardiogram showing a highly mobile mass in the distal aortic arch.

**Figure 2:**
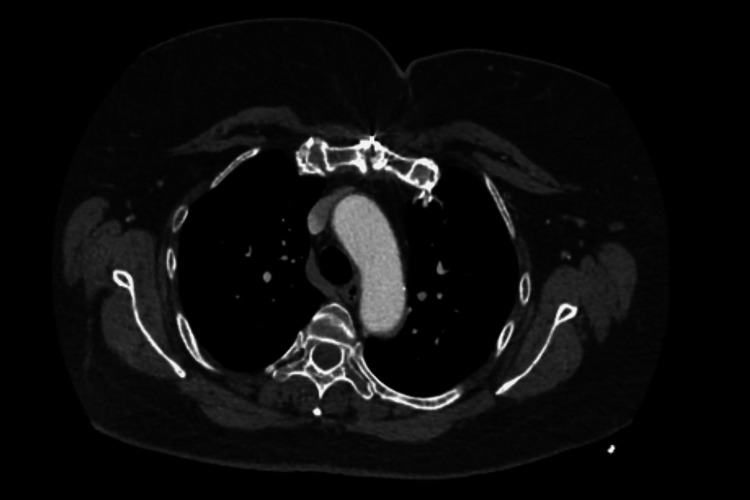
Follow-up CTA/aortogram three months following discharge showing total resolution of aortic arch thrombus. CTA: computed tomography angiogram

The patient was treated for her ongoing COPD exacerbation with the improvement of her respiratory symptoms. Her chest pain eventually resolved with intravenous morphine. Extensive workup for coagulopathies including protein C and S activity, antithrombin III levels, factor V (Leiden) mutation, prothrombin gene mutation, lupus anticoagulant profile, and phospholipid (cardiolipin) IgG/IgA/IgM was unremarkable. Cardiothoracic surgery was recommended against surgical intervention given the critical location of the thrombus and the patient’s recent history of CABG. A decision was made to start the patient on chronic anticoagulation and she was discharged on oral rivaroxaban 20 mg once daily. She was advised to seek immediate medical advice if she develops any symptoms or signs suggestive of peripheral, renal, or gastrointestinal ischemia. A follow-up chest CTA three months later showed total resolution of the thrombus (Figure 2). The patient's rivaroxaban was therefore discontinued. She continues to follow up with the outpatient cardiology clinic.

## Discussion

A floating aortic arch thrombus represents a rare incident with possible catastrophic sequelae. The pathophysiology remains unclear but possible predisposing factors include atherosclerosis, hypercoagulability disorders, malignancy, chronic inflammatory states, substance use, and chronic steroid use [3-6]. Despite this patient’s history of CAD, evidence of atherosclerosis was not present within the aortic arch on CTA, nor was there evidence of an underlying dissection or aneurysm. Furthermore, the patient's hypercoagulability workup was unremarkable.

Diagnosis of aortic arch thrombi remains a challenging task given that many patients remain asymptomatic before presenting with serious embolic events [7]. Hence, diagnosis is often established coincidentally on chest imaging modalities or echocardiographic evaluation [8]. This patient’s chest pain led to further workup that eliminated other differentials. An unremarkable electrocardiogram and normal troponin level ruled out myocardial ischemia. CTA did not show PE, nor an underlying aneurysm or dissection. CTAs are particularly useful in determining the location and extent of aortic thrombi [8]. Although transthoracic and TEEs are more useful in proximal aortic thrombi, echocardiography was valuable in evaluating the size and attachment of the thrombus, thus, aiding in the plan of management in this case [3].

Aortic arch thrombi can lead to grave consequences including embolization to the cerebral, peripheral, or visceral vasculature. Lower extremity arterial emboli are the most common, followed by the mesenteric and renal arteries. Cerebral and coronary emboli presenting with strokes or myocardial ischemia were reported in the literature as well [9,10]. Pedunculated and highly mobile lesions are more prone to embolism [11].

Treatment of aortic mural thrombi is largely based on clinical experience [6]. Surgical resection or endovascular depends on the location of adherence to the aortic wall and surgical candidacy [4,6,12]. The endovascular treatment provides a minimally invasive option with fewer complications, especially in patients who are at high risk for surgery or where conservative management was unsuccessful [4]. While systemic thrombolysis can be highly effective in dissolving mural thrombi, it poses an increased risk of distal embolic showers [13]. Anticoagulation with warfarin or direct oral anticoagulants (DOACs) is the mainstay of medical management in literature today [14-16]. However, guidelines regarding the type, dose, and duration of anticoagulation are yet to be established [17,18]. Moreover, although medical management alone has been shown to be successful, the incidence of persistent thrombi and recurrent embolism are reported to be much higher than in open surgical removal of thrombi [2,19,20].

## Conclusions

Intraluminal aortic arch thrombi should remain on the differentials list for incidental filling defects found on chest imaging. Early detection and management are of paramount importance in preventing serious complications. If surgical intervention is deemed unfeasible, anticoagulation with DOACs is an available and effective treatment modality.
